# Fine particulate matter constituents associated with emergency room visits for pediatric asthma: a time-stratified case–crossover study in an urban area

**DOI:** 10.1186/s12889-021-11636-5

**Published:** 2021-08-26

**Authors:** Yu-Ni Ho, Fu-Jen Cheng, Ming-Ta Tsai, Chih-Min Tsai, Po-Chun Chuang, Chi-Yung Cheng

**Affiliations:** 1 Department of Emergency Medicine, Kaohsiung Chang Gung Memorial Hospital, No.123, Dapi Rd, Niao-Sung Dist, Kaohsiung City, 833 Taiwan; 2 Chang Gung University College of Medicine, No.259, Wenhua 1st Road, Guishan District, Taoyuan City, 333 Taiwan; 3 Department of Pediatrics, Kaohsiung Chang Gung Memorial Hospital, No.123, Dapi Rd, Niao-Sung Dist, Kaohsiung City, 833 Taiwan; 4 Department of Computer Science and Engineering, National Sun Yat-sen University, No. 70, Lianhai Rd., Gushan Dist, Kaohsiung City, 804 Taiwan

**Keywords:** Particulate matter, Component, Air pollution, Pediatric, Asthma

## Abstract

**Background:**

Global asthma-related mortality tallies at around 2.5 million annually. Although asthma may be triggered or exacerbated by particulate matter (PM) exposure, studies investigating the relationship of PM and its components with emergency department (ED) visits for pediatric asthma are limited. This study aimed to estimate the impact of short-term exposure to PM constituents on ED visits for pediatric asthma.

**Methods:**

We retrospectively evaluated non-trauma patients aged younger than 17 years who visited the ED with a primary diagnosis of asthma. Further, measurements of PM with aerodynamic diameter of < 10 μm (PM_10_), PM with aerodynamic diameter of < 10 μm (PM_2.5_), and four PM_2.5_ components (i.e., nitrate (NO^3−^), sulfate (SO_4_^2−^), organic carbon (OC), and elemental carbon (EC)) were collected between 2007 and 2010 from southern particulate matter supersites. These included one core station and two satellite stations in Kaohsiung City, Taiwan. A time-stratified case-crossover study was conducted to analyze the hazard effect of PM.

**Results:**

Overall, 1597 patients were enrolled in our study. In the single-pollutant model, the estimated risk increase for pediatric asthma incidence on lag 3 were 14.7% [95% confidence interval (CI), 3.2–27.4%], 13.5% (95% CI, 3.3–24.6%), 14.8% (95% CI, 2.5–28.6%), and 19.8% (95% CI, 7.6–33.3%) per interquartile range increments in PM_2.5_, PM_10_, nitrate, and OC, respectively. In the two-pollutant models, OC remained significant after adjusting for PM_2.5_, PM_10_, and nitrate. During subgroup analysis, children were more vulnerable to PM_2.5_ and OC during cold days (< 26 °C, interaction *p* = 0.008 and 0.012, respectively).

**Conclusions:**

Both PM_2.5_ concentrations and its chemical constituents OC and nitrate are associated with ED visits for pediatric asthma. Among PM_2.5_ constituents, OC was most closely related to ED visits for pediatric asthma, and children are more vulnerable to PM_2.5_ and OC during cold days.

## Background

Asthma as a chronic inflammatory lung disease causes changeable airway hyper-responsiveness. It is a heterogeneous disease that varies widely in severity and clinical presentation, ranging from wheezing, dyspnea, and chest tightness to cough. An asthma attack could also lead to inconstant airflow obstruction and even death [1]. In 2016, more than 339 million people had asthma worldwide, and it accounted for more than 1000 deaths every day [2].

Epidemiological studies have reported that ambient air pollution are associated with poor health outcomes, including respiratory diseases [3, 4], cardiovascular diseases [5, 6], and mortality [7, 8]. In addition, exposure to air pollution is associated with airway inflammation and impairs lung function [9, 10]. Air pollutants are emitted from a range of sources, and thus identifying source-specific contributors that impact human health is important [11]. Exposure to air pollutants including particulate matter with aerodynamic diameter of < 10 μm (PM_10_) [10], particulate matter with aerodynamic diameter of < 2.5 μm (PM_2.5_) [12], sulfur dioxide (SO_2_) [13], and nitrogen dioxide (NO_2_), may induce or aggravate asthma. In children, higher PM_2.5_ exposure is associated with higher missed school days, hospitalization, and the frequency of asthma attack [14]. Among air pollutants, PM_2.5_ appears to have the greatest impact on the risk of asthma in children. After adjusting for NO_2_ and ozone (O_3_), PM_2.5_ was independently associated with asthma exacerbation [15], and the impact of PM_2.5_ remained statistically significant after adjusting for SO_2_ and NO_2_ [16]. This shows that although ambient PM_2.5_ exposure has an impact across all age groups, children are more sensitive to such exposure [12].

Target health outcomes vary by PM component. PM_2.5_ organic extracts mainly contain polycyclic aromatic hydrocarbons (PAHs), such as fluoranthene, rather than aqueous extracts, which mainly contain metals. A toxicological research showed that exposure to PM_2.5_ organic extracts caused a pro-inflammatory response in airway epithelial cells and affected the respiratory system [17]. Peng et al. demonstrated that among chemical constituents of PM_2.5_, ambient levels of organic carbon (OC) matter and elemental carbon (EC) were the strongest risk factors of emergency admissions [18]. Ostro et al. reported that exposure to components of PM_2.5_ (e.g., elemental and organ carbon, nitrate, sulfate, iron, potassium, and silicon) increased the risk of hospitalization for pediatric respiratory diseases such as bronchitis, asthma, and pneumonia [19].

Despite this adverse impact of air pollution on the pediatric population, limited studies have investigated the relationship of PM and its components with emergency department (ED) visits for pediatric asthma. As such, the present study aimed (1) to evaluate the correlation between short-term exposure to PM_2.5_ and its constitutes and the risk of pediatric asthma and (2) to determine the potential season and specific age groups who are more sensitive to the hazard effect of PM_2.5_ and its constitutes.

## Methods

### Study design and population

This retrospective observational study was conducted in a metropolitan tertiary medical center in Kaohsiung, Taiwan. The center receives an average of 72,000 ED visits per year. The subjects were non-trauma patients aged younger than 17 years [20] and who visited the ED with a primary diagnosis of asthma (International Classification of Diseases, Ninth Revision [ICD-9]: 493) between January 1, 2007 and December 31, 2010. Each episode of “asthma ED visit” was defined as “acute exacerbation of asthma with ED visit.” Medical records from the ED administrative database were reviewed by two trained emergency physicians. These included demographic factors including sex, age, address, and ED visiting time.

### Pollutant and meteorological data

**Fig. 1:**
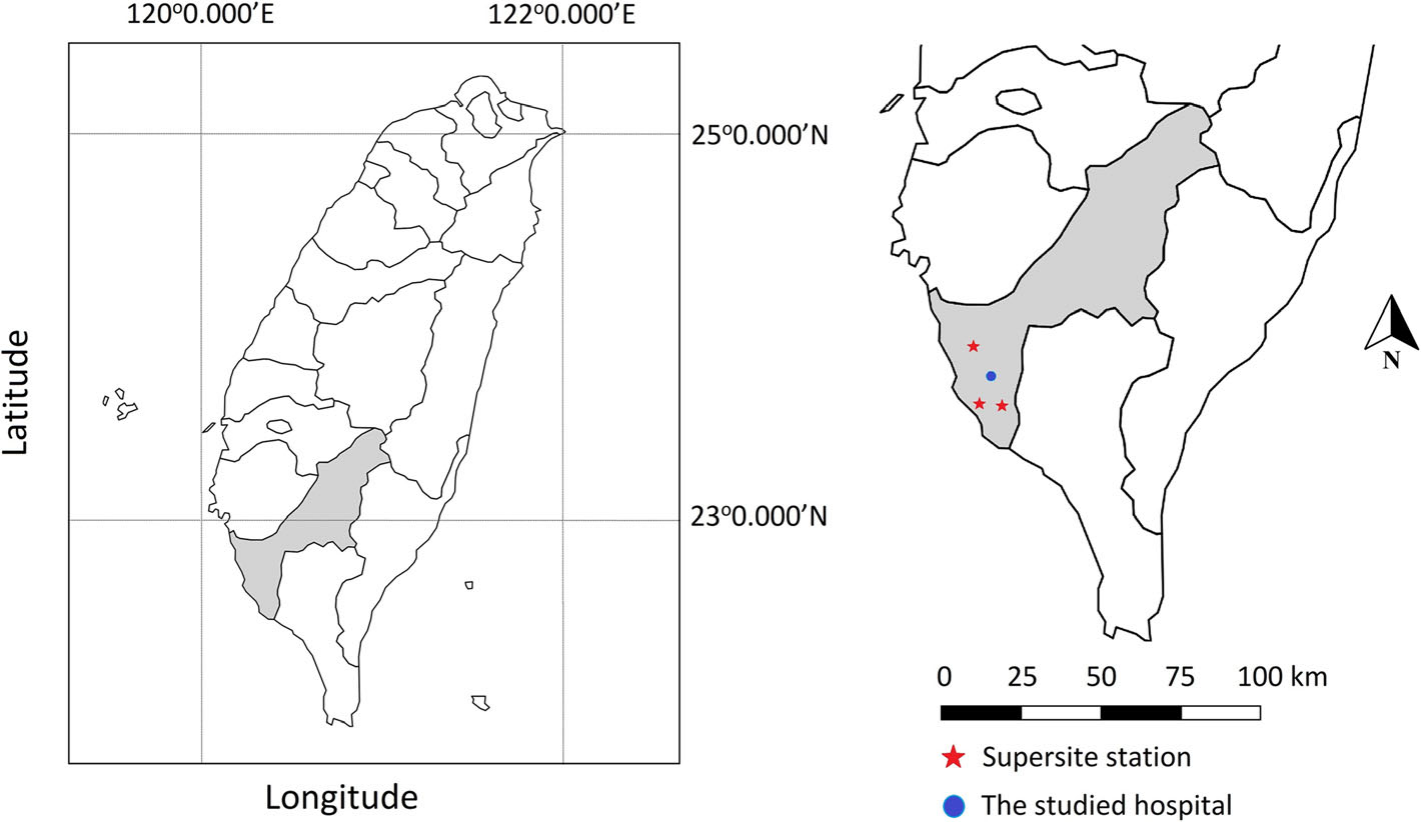
The locations of the three particulate matter supersites and the studied hospital in southwestern Taiwan. Note. The Taiwan map outline was adapted from https://webvectormaps.com/taiwan-map-outline-free-blank-vector-map/, which was licensed under the Creative Commons Attribution 4.0 International License. The locations of the three particulate matter supersites and the studied hospital in Kaohsiung was further created using Adobe Photoshop CC 2020

Air pollutant data and meteorological data from three particulate matter supersites located in Kaohsiung City were acquired (Fig. 1). These sites were established by the Taiwanese Environmental Protection Administration from 2005 to 2010. Hourly mass concentrations of PM_10_ and PM_2.5_ at these supersites are determined using the Rupprecht & Patashnick, Co. Series 1400a Tapered Element Oscillation Microbalance particle monitor; OC and EC using the Series 5400 Ambient Carbon Particulate Monitor; nitrate using the Series 8400 N Particulate Nitrate Monitor; and sulfate using the Series 8400S Particulate Sulfate Monitor (Environmental Protection Administration Executive Yuan, R.O.C., Taipei 2010).

Hourly mass concentrations of PM_10_, PM_2.5_, and the four PM_2.5_ components (i.e., nitrate (NO_3_^−^), sulfate (SO_4_^2−^), OC, and EC) during the study period were collected. We collected the daily average of PM and its components from each monitoring supersite. The patient addresses were reviewed, and the 24-h average levels of above ambient air pollutants from the nearest monitoring station were computed. The 24-h recordings of mean temperature and mean humidity from the monitoring stations were also collected.

### Statistical analysis

A time-stratified case-crossover study, which is an equivalent to Poisson time series regression models, was conducted to analyze asthma events, air pollutants, and meteorological data [21, 22]. This study design is a special type of case-control study. We compared subjects between case periods and control periods. Using time stratification to select the referent days, we chose the days falling on the same day of the week (1 case day with 3–4 control days), which is in the same month of the same year as the case period. The self-matching time stratification strategy was performed to adjust the effects of long-term trends, seasonal effects, and day of the week [23]. The day of pediatric asthma ED visit was set as lag 0, the day before the pediatric asthma event was lag 1, and the day before lag 1 was lag 2, and so forth. We compared the levels of air pollution between the case period and all the referent days. Then, the impact of environment variables on pediatric asthma from lags 0 to 3 was investigated. Conditional logistic regression was used to estimate the odds ratios (ORs) and 95% confidence intervals (CIs) for the percent change in odds of ED visits for pediatric asthma cases with PM_2.5_ mass and its constituents. To identify the most susceptible groups, subgroup analyses was performed using age, sex, and weather conditions. Temperature and relative humidity were included as confounding factors in our model. Potential non-linear effects between temperature, humidity, and pediatric asthma were determined using Akaike’s information criterion (AIC) [24]. This step was performed by using SAS macro “lgtphcurv9,” which implements natural cubic spline methodology to fit potential non-linear response curves in logistic regression models for case-control studies [25]. The AIC value was lower in the linear model (4399.806) than in the spline model (4401.630) for temperature. Further, the test of curvature (nonlinear response) was nonsignificant (*p* = 0.068). Meanwhile, the AIC value was lower in the spline model (4399.921) than that in the linear model (4400.026) for humidity, and the test of curvature was significant (*p* = 0.039). According to the results of AIC, the spline model was used to create a five categorical variable in accordance with knots for humidity [26]. Consequently, we selected the linear model for temperature and spline model for humidity as confounding factors in conditional logistic regression analysis. The ORs were computed using interquartile range (IQR) increments in PM_10_, PM_2.5_, and PM_2.5_ constituents. All statistical analyses were performed using SAS, version 9.3. All tests were two tailed, and *P* < 0.05 was considered statistically significant.

## Results

A total of 1712 pediatric asthma patients visited the ED within the 4-year study period. Of them, 115 patients were excluded from the analysis because they did not live in Kaohsiung City. Thus, 1597 patients with a mean age of 6.1 ± 3.5 years were included in our study. The patient characteristics are summarized in Table 1. There were 1073 (67.2%) male patients. Majority of the ED visits due to asthma were during the cold season (October to March) (60.8%). The most frequent comorbidities were hypertension (11 children) and cerebral palsy (5 children). One child had history of epilepsy, one child had history of malignancy, and one had history of chronic respiratory failure.

**Table 1:** Characteristics of the cases (*n* = 1597)

Characteristic	Number	%
Age (mean ± SD)	6.1 ± 3.5	
Male sex	1073	67.2
Cold season	971	60.8
Cold days (< 26.0 °C)	879	55.1

The daily average temperature, humidity, and mean concentrations of air pollutants in Kaohsiung City during the study period are shown in Table 2. The average PM_2.5_ concentration was 32.7 ± 15.9 μg/m^3^. Among the components of PM_2.5_, nitrate, sulfate, OC and EC accounted for 4.4 ± 3.3 μg/m^3^ (13.5% of PM_2.5_ mass), 9.4 ± 4.8 μg/m^3^ (28.7% of PM_2.5_ mass), 8.2 ± 3.7 μg/m^3^ (25.1% of PM_2.5_ mass), and 2.1 ± 0.9 μg/m^3^ (6.4% of PM_2.5_ mass), respectively.

**Table 2:** Summarized statistics for meteorology and air pollution in Kaohsiung, 2007–2010

Percentiles							
	Minimum	25%	50%	75%	Maximum	Mean	IQR
PM_2.5_ (μg/m^3^)	6.9	18.9	31.6	43.0	119.5	32.7	24.1
PM_10_ (μg/m^3^)	10.7	29.7	46.6	66.9	449.5	50.3	37.2
Nitrate (μg/m^3^)	0.3	1.4	3.9	6.6	20.7	4.4	5.2
Sulfate (μg/m^3^)	1.1	5.6	9.1	12.5	33.7	9.4	6.9
Organic carbon (μg/m^3^)	1.4	5.4	7.5	10.6	27.8	8.2	5.2
Elemental carbon (μg/m^3^)	0.5	1.5	2.0	2.6	16.5	2.1	1.1
Temperature (°C)	13.4	22.6	26.5	28.8	31.6	25.5	6.2
Humidity (%)	44.0	69.0	73.4	77.3	95.3	73.2	8.3

The missing data for all monitor stations were less than 1%

**Fig. 2:**
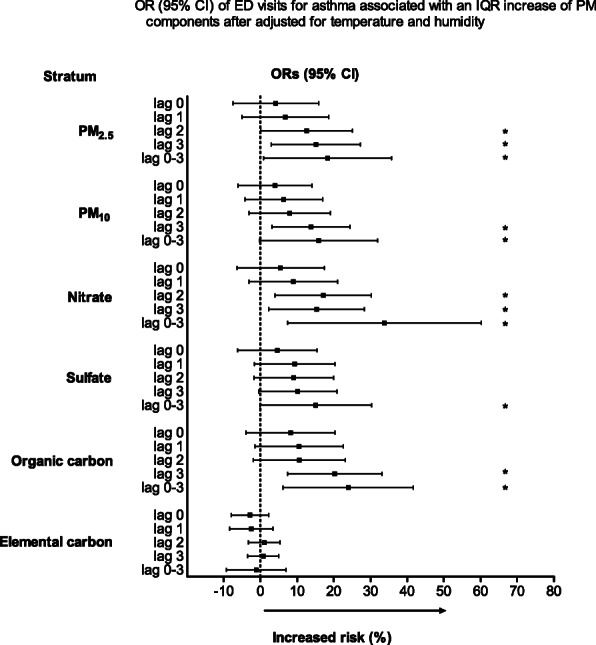
Odds ratios (ORs) and 95% confidence intervals (CIs) for pediatric asthma ED visits. The values are shown according to IQR increments in PM_2.5_ and the levels of its constituents, with adjustment for temperature and humidity. ED, emergency department; IQR, interquartile range

The Spearman correlation coefficients for the air pollutants and weather conditions are listed in Table 3. PM_2.5_ was highly correlated with PM_10_ (r = 0.909, *p* < 0.001), sulfate (r = 0.908, p < 0.001), and OC (r = 0.822, p < 0.001) and moderately correlated with nitrate (r = 0.793, p < 0.001) and EC (r = 0.699, p < 0.001). The continual estimates of PM_2.5_ and its constituents according to the percent change in odds of ED visits for pediatric asthma are shown in Fig. 2. On lag 2, the percent change in odds of ED visits for pediatric asthma was 12.1% (95% CI, 0.3–25.3%) and 16.6% (95% CI, 4.2–30.5%) per IQR increment in PM_2.5_ and nitrate, respectively. On lag 3, the percent change in odds of ED visits for pediatric asthma were 14.7% (95% CI, 3.2–27.4%), 13.5% (95% CI, 3.3–24.6%), 14.8% (95% CI, 2.5–28.6%), and 19.8% (95% CI, 7.6–33.3%) per IQR increment in PM_2.5_, PM_10_, nitrate, and OC, respectively. Meanwhile, an IQR increase in sulfate and EC levels were not significantly associated with asthma. For the 4-day moving average (lag 0–3) of each particulate pollutant, the percent change in odds of ED visits for pediatric asthma were 17.4% (95% CI, 1.3–36.2%), 15.1% (95% CI, 0.2–32.3%), 32.1% (95% CI, 8.3–61.0%), 14.4 (95% CI, 0.2–30.6%), and 23.1 (95% CI, 6.6–42.1%) per IQR increment in PM_2.5_, PM_10_, nitrate, sulfate, and OC, respectively.

**Table 3:** Spearman correlation coefficients between air pollutants and weather conditions during the 4-year study period

	PM_**10**_	PM_**2.5**_	Nitrate	Sulfate	Organic carbon	Elemental carbon	Temperature	Humidity
PM_10_	1.000	0.909	0.669	0.774	0.731	0.568	−0.493	−0.410
PM_2.5_		1.000	0.793	0.908	0.822	0.699	−0.504	− 0.406
Nitrate			1.000	0.680	0.833	0.643	−0.580	−0.269
Sulfate				1.000	0.673	0.592	−0.403	−0.359
Organic carbon					1.000	0.732	−0.536	−0.377
Elemental carbon						1.000	−0.376	−0.277
Temperature							1.000	0.315
Humidity								1.000

To determine the independent effect of each pollutant, a two-pollutant model was created to estimate the contamination influence on pediatric asthma ED visits. To assess the responsible pollutant for the observed contamination effects, various combinations of two different pollutants were fitted in the two-pollutant model according to the finding obtained from the single-pollutant models. Given that PM_2.5_ is a part of PM_10_, we used coarse particulate matter (PM_c_), which is particulate matter with an aerodynamic diameter of 2.5–10 μm, to replace PM_10_ in the two-pollutant model. The results of the two-pollutant model are summarized in Table 4. After adjustment for PM_2.5_ (OR = 1.168, 95% CI: 1.016–1.342), PM_c_ (OR = 1.174, 95% CI: 1.053–1.309), and nitrate (OR = 1.175, 95% CI: 1.026–1.345), an IQR increase in OC was found to be significantly associated with ED visits for pediatric asthma in the two-pollutant model.

**Table 4:** Emergency department visits for each interquartile range change in the two-pollutant models

	OR (95% CI) of asthma Adjusted for temperature, humidity, and pollutant			
Single-pollutant model	Adjusted PM_2.5_	Adjusted PM_10_	Adjusted Nitrate	Adjusted Organic carbon
PM_2.5_		1.064 (0.889–1.274)	1.099 (0.965–1.251)	1.040 (0.906–1.193)
PM_10_	1.085 (0.925–1.272)		1.102 (0.995–1.220)	1.068 (0.959–1.188)
Nitrate	1.083 (0.942–1.245)	1.096 (0.969–1.240)		1.068 (0.895–1.194)
Organic carbon	1.168 (1.016–1.342)	1.155 (1.022–1.305)	1.175 (1.026–1.345)	

**Fig. 3:**
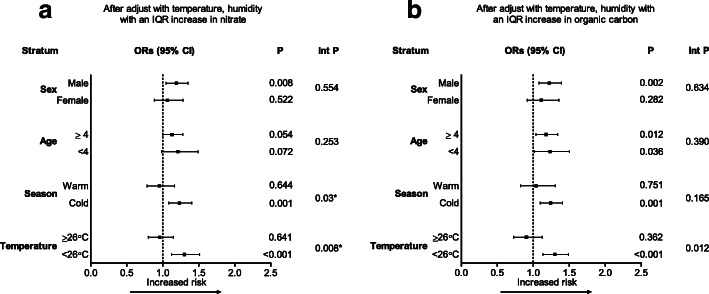
Odds ratios (ORs) for pediatric asthma ED visits according to IQR increments. **a** nitrate and **b** organic carbon on lag 3 after adjustment for temperature and humidity. *p < 0.05. Int p, interaction *p*-value

Based on different seasons and demographic factors on lag 3, we conducted a stratified analysis to examine the impact of PM_2.5_ and OC on pediatric asthma (Fig. 3). After adjusting for temperature and humidity, the percent change in odds of ED visits for pediatric asthma after exposure to PM_2.5_ was higher during cold season (inter *p* = 0.03) and cold days (< 26 °C, inter *p* = 0.008; Fig. 3a). Further, patients were more vulnerable to the adverse effects of OC on asthma during cold days (< 26 °C, interaction *p* = 0.012; Fig. 3b).

## Discussion

Asthma may be triggered or exacerbated by PM exposure, but studies investigating the relationship of PM and its components with ED visits for pediatric asthma are scarce. The present study found that PM_2.5_ and its constituents nitrate and OC may play an essential role in ED visits for pediatric asthma in Kaohsiung, Taiwan. Furthermore, OC exerted robust effects after adjusting for PM_2.5_, PM_10_, and nitrate. Children were more vulnerable to PM_2.5_ and OC during cold days.

PM exposure increases the risk for cardiovascular events [5, 6] and respiratory disease [3, 4, 12, 14]. Children are highly vulnerable to the adverse effects of air pollution, particularly to respiratory diseases [12]. Maternal air pollution exposure was reported to have an adverse impact on the growth, development, and function of the placenta. Exposure to particle pollution is even associated with adverse effects in the utero [27]. Prenatal PM_2.5_ exposure was proven to increase the risk of childhood asthma [28]. Previous studies also found significantly positive associations of PM_2.5_ exposure with ED visits for asthma and respiratory morbidity in children [29, 30]. In a prospective study of 1759 children in Southern California, Gauderman et al. found that air pollution limits lung development in children aged 10 to 18 years [9]. Delfino et al. also found a positive association between lung function deficits in schoolchildren with persistent asthma and increased ambient air pollution exposures to NO_2_ and PM_2.5_ [31]. Similarly, our study also shows that exposure to PM_2.5_ and its constituents OC and nitrate is associated with ED visits for pediatric asthma.

PM is composed of various sizes and chemical mixture particles, and target health outcomes vary according to these components. Hwang, et al. indicated that lagged SO_4_^2−^ was associated with a higher number of ER visits for respiratory diseases, while NH4+ was the most significant influencing factor of cardiovascular ER visits, followed by OC, SO_4_^2−^, NO_3_^−^, and EC. Emergency room visits for asthma is more closely related to the estimated effects of NO_3_^−^ [32]. However, in the present study, among PM_2.5_ constituents, it was OC that was most closely related to ED visits for pediatric asthma. One possible reason for this discrepant result was that Hwang’s study enrolled the entire population of pediatric and adult patients. Meanwhile, we only included pediatric patients aged less than 17 years. Susceptibility to PM and its constituents may vary by age, which can then in turn cause variations in the accuracy of inflammatory biomarkers and varying health effects [33]. Another possible reason is that the study population in Hwang’s study was from two adjacent cities that were different from where the monitoring station located. As such, there might have been measurement errors due to the relatively long distance. In contrast, the present study only enrolled patients in a single city where the particulate matter supersite monitor station was located. Furthermore, PM_2.5_ was emitted from various sources including factories, vehicles, and windblown dust. The diverse origin and geographical features may account for the differences in characteristic toxicity. The difference in source of PM_2.5_ exposure could, in part, explain the distinct association between PM_2.5_ exposure and asthma in children [34].

Exposure to PM may cause airway hyper-responsiveness and asthma. Murine asthma models have shown that exposure to PM increased neutrophil infiltration by increasing tumor necrosis factor alpha and interferon gamma excretion. Further, it also increased allergic immune responses by increasing the production of T helper type 2 cell-related cytokines including interleukin-5 and interleukin-13 [35]. In addition, a mice experiment found that PM exposure could trigger the release of inflammatory cytokines of macrophages in the lung [36]. Liu et al. reported that PM_2.5_ exposure significantly increased oxidative stress in the airways and impaired the function of the small airways in asthmatic children [37]. Using a nationally representative sample involving 3531 individuals aged 6 to 79 years, Cakmak et al. demonstrated that exposure to polycyclic aromatic hydrocarbons was related to lower one-second forced expiratory volume and forced vital capacity and poor respiratory outcomes [38]. Compared to the water extracts of PM_2.5_, exposure to the organic extracts is more likely to increase inflammatory response in the lung and liver [39]. Our findings indicated that among PM_2.5_ components, OC had the strongest impact. This result may indicate that diverse PM_2.5_ components and sources play a significant role in the adverse of pollution on human health.

The hazardous effect of air pollutants seems to vary by season. Cheng et al. implied higher levels of PM enhance the risk of hospital admissions for respiratory disease on cool days [40]. Huang et al. also showed that short-term exposure to PM was correlated with increased hospitalizations for cerebrovascular accident on warm days [41]. Ueda et al. found higher associations between PM_2.5_ mass and cardiovascular mortality during fall, while associations with respiratory mortality were stronger in spring [42]. Hwang et al. revealed that PM_2.5_ constituents were more closely related to ED visits for children with asthma in the hot season [32]. Meanwhile, Guo et al. reported that the prevalence of asthma was positively correlated with non-summer temperature and winter humidity [43]. In our study, children were more vulnerable to PM_2.5_ (inter *p* = 0.019) and OC (inter *p* = 0.012) during cold days. The seasonal difference between these studies support that the influence of air pollution on pediatric asthma may also be associated with weather, climate changes, and geographical features [44]. The correlation of air pollutants to temperature and humidity may imply that their composition and toxicity could be enhanced in certain conditions. One study showed that weather systems modified the distribution and concentration of PM_2.5_ mass [45]. Meteorological factors and atmospheric pollution have synergistic effects, and the combination of environmental factors is likely to influence the degree of asthma attack [46].

### Study limitations

Certain limitations should be noted in the study. First, the study was conducted in a single tertiary medical center from a coastal industrial city. Thus, the findings may not be generalizable to other regions considering the difference in pollution source, meteorological characteristics, and race. Next, we only included children who were more severe or poorly controlled asthma with ED visit. We did not include children who were treated in outpatient department settings. In addition, other factors such as sociodemographic conditions may influence the ER visits for asthma. Lastly, use of protective equipment such as air purifiers and face mask may reduce exposure to pollutants. To obtain more accurate effect estimates, further studies should include more regions, larger medical records, and precise measurement of individual exposure despite personal protective equipment.

## Conclusions

PM_2.5_, PM_10_, nitrate, and OC may play an essential role in ED visits for pediatric asthma in Kaohsiung, Taiwan. The effects of OC were robust after adjusting for PM_2.5_, PM_10_, and nitrate, especially during cold days.

## Data Availability

Air pollutant data and meteorological data used and analyzed were acquired from https://airtw.epa.gov.tw/, which is an open public access. The datasets of patients used and analyzed during the current study were acquired from Kaohsiung Chang Gung Memorial Hospital. The datasets were available from the corresponding author on reasonable request.

## Abbreviations

*AIC*: Akaike’s information criterion
*CI*: Confidence interval
*ED*: Emergency department
*IQR*: Interquartile range
*NO*_*2*_: Nitrogen dioxide
*ORs*: Odds ratios
*OC*: Organic carbon
*EC*: Elemental carbon
*PM*: Particulate matter
*PM*_*10*_: Particulate matter with aerodynamic diameter of < 10 μm
*PM*_*2*.5_: Particulate matter with aerodynamic diameter of < 2.5 μm
*SO*_*2*_: Sulfur dioxide

## References

[1] Dwyer-Lindgren L, Bertozzi-Villa A, Stubbs RW, Morozoff C, Shirude S, Naghavi M, Mokdad AH, Murray CJL (2017). Trends and patterns of differences in chronic respiratory disease mortality among US counties, 1980-2014. JAMA..

[2] GBD 2016 Disease and Injury Incidence and Prevalence Collaborators (2017). Global, regional, and national incidence, prevalence, and years lived with disability for 328 diseases and injuries for 195 countries, 1990–2016: a systematic analysis for the Global Burden of Disease Study 2016. Lancet.

[3] Cheng FJ, Lee KH, Lee CW, Hsu PC (2019). Association between particulate matter air pollution and hospital emergency room visits for pneumonia with septicemia: a retrospective analysis. Aerosol Air Qual Res.

[4] Trasande L, Thurston GD (2005). The role of air pollution in asthma and other pediatric morbidities. J Allergy Clin Immunol.

[5] Weichenthal S, Kulka R, Lavigne E, van Rijswijk D, Brauer M, Villeneuve PJ, Stieb D, Joseph L, Burnett RT (2017). Biomass burning as a source of ambient fine particulate air pollution and acute myocardial infarction. Epidemiology..

[6] Pan HY, Cheung SM, Chen FC, Wu KH, Cheng SY, Chuang PC, Cheng FJ (2019). Short-term effects of ambient air pollution on ST-elevation myocardial infarction events: are there potentially susceptible groups?. Int J Environ Res Public Health.

[7] Kim TY, Kim H, Yi SM, Cheong JP, Heo J (2018). Short-term effects of ambient PM2.5 and PM2.5-10 on mortality in major cities of Korea. Aerosol Air Qual Res.

[8] Di Q, Dai L, Wang Y, Zanobetti A, Choirat C, Schwartz JD (2017). Association of short-term exposure to air pollution with mortality in older adults. JAMA..

[9] Gauderman WJ, Avol E, Gilliland F, Vora H, Thomas D, Berhane K, McConnell R, Kuenzli N, Lurmann F, Rappaport E, Margolis H, Bates D, Peters J (2004). The effect of air pollution on lung development from 10 to 18 years of age. N Engl J Med.

[10] Donaldson K, Gilmour MI, MacNee W (2000). Asthma and PM10. Respir Res.

[11] Heal MR, Kumar P, Harrison RM (2012). Particles, air quality, policy and health. Chem Soc Rev.

[12] Fan J, Li S, Fan C, Bai Z, Yang K (2016). The impact of PM2.5 on asthma emergency department visits: a systematic review and meta-analysis. Environ Sci Pollut Res Int.

[13] Andersson E, Knutsson A, Hagberg S, Nilsson T, Karlsson B, Alfredsson L, Torén K (2006). Incidence of asthma among workers exposed to Sulphur dioxide and other irritant gases. Eur Respir J.

[14] O'Connor GT, Neas L, Vaughn B, Kattan M, Mitchell H, Crain EF (2008). Acute respiratory health effects of air pollution on children with asthma in US inner cities. J Allergy Clin Immunol.

[15] Bouazza N, Foissac F, Urien S, Guedj R, Carbajal R, Tréluyer JM, Chappuy H (2018). Fine particulate pollution and asthma exacerbations. Arch Dis Child.

[16] Ma Y, Yu Z, Jiao H, Zhang Y, Ma B, Wang F, Zhou J (2019). Short-term effect of PM2.5 on pediatric asthma incidence in Shanghai, China. Environ Sci Pollut Res Int.

[17] Honda A, Fukushima W, Oishi M, Tsuji K, Sawahara T, Hayashi T (2017). Effects of components of PM2.5 collected in Japan on the respiratory and immune systems. Int J Toxicol.

[18] Peng RD, Bell ML, Geyh AS, McDermott A, Zeger SL, Samet JM, Dominici F (2009). Emergency admissions for cardiovascular and respiratory diseases and the chemical composition of fine particle air pollution. Environ Health Perspect.

[19] Ostro B, Roth L, Malig B, Marty M (2009). The effects of fine particle components on respiratory hospital admissions in children. Environ Health Perspect.

[20] Akinbami LJ, Simon AE, Rossen LM (2016). Changing trends in asthma prevalence among children. Pediatrics..

[21] Marshall RJ, Jackson RT (1993). Analysis of case-crossover designs. Stat Med.

[22] Mittleman MA, Maclure M, Robins JM (1995). Control sampling strategies for case-crossover studies: an assessment of relative efficiency. Am J Epidemiol.

[23] Peng RD, Dominici F, Pastor-Barriuso R, Zeger SL, Samet JM (2005). Seasonal analyses of air pollution and mortality in 100 US cities. Am J Epidemiol.

[24] Aho K, Derryberry D, Peterson T (2014). Model selection for ecologists: the worldviews of AIC and BIC. Ecology..

[25] Li R, Hertzmark E, Louie M, Chen L, Spiegelman D (2011). The SAS LGTPHCURV9 Macro.

[26] DeVries R, Kriebel D, Sama S (2016). Low level air pollution and exacerbation of existing copd: a case crossover analysis. Environ Health.

[27] Laurent O, Hu J, Li L, Kleeman MJ, Bartell SM, Cockburn M, Escobedo L, Wu J (2016). A statewide nested case-control study of preterm birth and air pollution by source and composition: California, 2001-2008. Environ Health Perspect.

[28] Lee A, Hsu HHL, Chiu YHM, Bose S, Rosa MJ, Kloog I (2018). Prenatal fine particulate exposure and early childhood asthma: effect of maternal stress and fetal sex. J Allergy Clin Immunol.

[29] Farhat SCL, Paulo RLP, Shimoda TM, Conceição GMN, Lin CA, Braga ALF (2005). Effect of air pollution on pediatric respiratory emergency room visits and hospital admissions. Braz J Med Biol Res.

[30] Xiao Q, Liu Y, Mulholland JA, Russell AG, Darrow LA, Tolbert PE (2016). Pediatric emergency department visits and ambient Air pollution in the U.S. State of Georgia: a case-crossover study. Environ Health.

[31] Delfino RJ, Staimer S, Tjoa T, Gillen D, Kleinman MT, Sioutas C (2008). Personal and ambient air pollution exposures and lung function decrements in children with asthma. Environ Health Perspect.

[32] Hwang SL, Lin YC, Lin CM, Hsiao KY (2017). Effects of fine particulate matter and its constituents on emergency room visits for asthma in southern Taiwan during 2008-2010: a population-based study. Environ Sci Pollut Res Int.

[33] Hassanvand MS, Naddafi K, Kashani H, Faridi S, Kunzli N, Nabizadeh R, Momeniha F, Gholampour A, Arhami M, Zare A, Pourpak Z, Hoseini M, Yunesian M (2017). Short-term effects of particle size fractions on circulating biomarkers of inflammation in a panel of elderly subjects and healthy young adults. Environ Pollut.

[34] Habre R, Moshier E, Castro W, Nath A, Grunin A, Rohr A, Godbold J, Schachter N, Kattan M, Coull B, Koutrakis P (2014). The effects of PM2.5 and its components from indoor and outdoor sources on cough and wheeze symptoms in asthmatic children. J Expo Sci Environ Epidemiol.

[35] Huang KL, Liu SY, Chou CCK, Lee YH, Cheng TJ (2017). The effect of size-segregated ambient particulate matter on Th1/Th2-like immune responses in mice. PLoS One.

[36] Marchini T, Wolf D, Michel NA, Mauler M, Dufner B, Hoppe N, Beckert J, Jäckel M, Magnani N, Duerschmied D, Tasat D, Alvarez S, Reinöhl J, von zur Muhlen C, Idzko M, Bode C, Hilgendorf I, Evelson P, Zirlik A (2016). Acute exposure to air pollution particulate matter aggravates experimental myocardial infarction in mice by potentiating cytokine secretion from lung macrophages. Basic Res Cardiol.

[37] Liu L, Poon R, Chen L, Frescura AM, Montuschi P, Ciabattoni G (2009). Acute effects of air pollution on pulmonary function, airway inflammation, and oxidative stress in asthmatic children. Environ Health Perspect.

[38] Cakmak S, Hebbern C, Cakmak JD, Dales RE (2017). The influence of polycyclic aromatic hydrocarbons on lung function in a representative sample of the Canadian population. Environ Pollut.

[39] Pardo M, Xu F, Qiu X, Zhu T, Rudich Y (2018). Seasonal variations in fine particle composition from Beijing prompt oxidative stress response in mouse lung and liver. Sci Total Environ.

[40] Cheng MH, Chiu HF, Yang CY (2015). Coarse particulate air pollution associated with increased risk of hospital admissions for respiratory diseases in a tropical city, Kaohsiung, Taiwan. Int J Environ Res Public Health.

[41] Huang F, Luo Y, Guo Y, Tao L, Xu Q, Wang C (2016). Particulate matter and hospital admissions for stroke in Beijing, China: modification effects by ambient temperature. J Am Heart Assoc.

[42] Ueda K, Yamagami M, Ikemori F, Hisatsune K, Nitta H (2016). Associations between fine particulate matter components and daily mortality in Nagoya, Japan. J Epidemiol.

[43] Guo YL, Lin YC, Sung FC, Huang SL, Ko YC, Lai JS, Su HJ, Shaw CK, Lin RS, Dockery DW (1999). Climate, traffic-related air pollutants, and asthma prevalence in middle-school children in Taiwan. Environ Health Perspect.

[44] Kinney PL (2018). Interactions of climate change, air pollution, and human health. Curr Environ Health Rep.

[45] Poole JA, Barnes CS, Demain JG, Bernstein JA, Padukudru MA, Sheehan WJ, Fogelbach GG, Wedner J, Codina R, Levetin E, Cohn JR, Kagen S, Portnoy JM, Nel AE (2019). Impact of weather and climate change with indoor and outdoor air quality in asthma: a work group report of the AAAAI environmental exposure and respiratory health committee. J Allergy Clin Immunol.

[46] Huang CH, Lin HC, Tsai CD, Huang HK, Lian IB, Chang CC (2017). The interaction effects of meteorological factors and air pollution on the development of acute coronary syndrome. Sci Rep.

